# *“Is this the GVHD?”* A qualitative exploration of quality of life issues in individuals with graft-versus-host disease following allogeneic stem cell transplant and their experiences of a specialist multidisciplinary bone marrow transplant service

**DOI:** 10.1186/s12955-020-01651-2

**Published:** 2021-01-07

**Authors:** Isabella de Vere Hunt, James M. Kilgour, Robert Danby, Andy Peniket, Rubeta N. Matin

**Affiliations:** 1 Department of Dermatology, Oxford University Hospitals NHS Foundation Trust, Oxford, UK; 2 Department of Dermatology, Stanford University, Discovery Hall - D143, 455 Broadway, Redwood City, CA 94063 USA; 3 Department of Haematology, Oxford University Hospitals NHS Foundation Trust, Oxford, UK

**Keywords:** Graft-versus-host disease, GVHD, Allogenic stem cell transplant, Bone marrow transplant, Haematology, Qualitative research, Quality of life, Experience of service provision, Multidisciplinary care

## Abstract

**Background:**

Graft-versus-host disease (GVHD) is a significant cause of morbidity and mortality following allogeneic stem cell transplantation. These patients face unique challenges due to the complexity of GVHD which can affect multiple organ systems, and the toxicity of treatments. Despite the known impact on quality of life (QOL), qualitative data within the bone marrow transplantation (BMT) literature is rare, and there has been no qualitative work exploring patient experience of specialist healthcare provision for GVHD in the United Kingdom.

**Methods:**

We conducted a primary explorative qualitative study of the experience of QOL issues and multidisciplinary care in patients with chronic GVHD following allogeneic stem cell transplantation. Eight patients were identified using convenience sampling from specialist BMT outpatient clinics. Following consent, patients were interviewed individually via telephone. Transcripts of interviews were analyzed using an inductive thematic approach.

**Results:**

Mean participant age was 61-years-old (range 45–68), with a mean time post-transplant of 3 years at time of interview (range 3 months–15 years). Five key QOL themes were identified: (1) *‘Restricted as to what I can do’*; (2) Troubling symptoms—*‘you can sort of get GVHD anywhere’*; (3) Confusion/uncertainty over GVHD symptoms—*‘Is this the GVHD?’*; (4) Unpredictable course and uncertainty about the future; and (5) Adapting to the sick role. In addition, four themes related to experience of service provision were identified: (1) personal care and close relationship with BMT nurses; (2) efficiency versus long waits—*‘On the case straight away’*; (3) information provision—*‘went into it with a bit of a rosy view’*; and (4) the role of support groups.

**Conclusions:**

These qualitative data reflect the heterogeneity of experiences of the GVHD patient population, reflecting the need for a flexible and nuanced approach to patient care with emphasis on comprehensive information provision. We have identified the key role that BMT specialist nurses within the multidisciplinary team play in supporting patients. We advocate future research should focus on ways to meet the complex needs of this patient group and ensure that the personal care and close relationships are not lost in service redesigns embracing remote consultations.

## Introduction

Graft-versus-host disease (GVHD) is a major complication of haematopoietic stem cell transplantation (HSCT), and a cause of significant morbidity and mortality in this patient group. GVHD is a systemic disease, able to cause pleiotropic effects in most organ systems of the body occurring at any time post-transplant [1, 2]. Both acute (aGVHD) and chronic GVHD (cGVHD) following transplantation can have a profound and lasting impact on health-related quality of life (QOL) of allogeneic stem cell transplant recipients [3]. cGVHD can persist for many years resulting in reduced functional status, inability to resume normal activities of daily living, including return to employment, and significant symptom burden [4–6]. Management of these patients relies on using validated and sensitive patient-reported outcome instruments (PROMs), focusing on QOL and symptom burden, to better monitor disease progression and treatment response. However, our recent systematic review of PROMs in this patient group revealed that there are limited qualitative data in the bone marrow transplant literature exploring QOL themes [7], with only 11 qualitative articles focussing on GVHD [8–18].

The polymorphic multi-systemic nature of GVHD and poor evidence-base for treatment options makes the diagnosis and effective management for GVHD very challenging, and the British Committee for Standards in Haematology GVHD guidelines recommend organ‐specific management and supportive care [19]. Given the diverse range of symptoms and affected organ systems that these patients experience, multidisciplinary specialist care clinics at tertiary centres have been established in the U.K. [20]. However, the patient experience of these clinics and their subjective impact on patients requires consideration. In previous qualitative work in the U.S., survivors of HSCT have expressed a preference for care from specialists, feeling that other providers did not understand the complexities of their condition [13]. Furthermore, U.S. focus groups identified concerns whereby patients felt let-down and *‘left on their own’* to deal with their cGVHD care post-HSCT [17, 18]. To our knowledge, there has been no qualitative work exploring patient experience of healthcare provision for GVHD conducted in the U.K.

The overall aim of this study was to optimize management of GVHD patients following HSCT using a cross-sectional qualitative exploration of patient perspectives of their health-related QOL (HRQOL) and experiences of service provision in a multidisciplinary specialist care clinic. The following research questions were addressed:

1. What are the key QOL issues that affect patients with GVHD following HSCT?
2. What is the patient experience of the multidisciplinary service provision for GVHD?

## Methods

Ethical approval for human subject research was granted by the North West—Preston NHS Research Ethics committee in April 2019 (reference 19/NW/0198). Funding for this research was received from the Oxfordshire Health Services Research Committee (grant reference 1304). The overall research design is a thematic analysis of in-depth, semi-structured patient interviews. Thematic analysis is a key method for identifying, analysing and reporting patterns within qualitative data [21].

### Recruitment

Participants were identified from outpatient BMT clinics held at our tertiary care, university-affiliated cancer centre. Eligible participants were adults with a confirmed diagnosis of acute or chronic GVHD following allogeneic stem cell transplant. Participants with current active malignancy, receiving inpatient care or unable or unwilling to consent were excluded. Eligibility was screened by a member of the usual clinical team (RM) who had access to the clinical notes and was able to confirm the diagnosis of GVHD. All eligible patients attending the clinic over 3 months were invited to participate in the study. A written participant information sheet was provided and signed written consent forms were completed by patients who agreed to participate. All participant responses were anonymised.

### Interviews

The qualitative investigator (JK) conducted in-depth, semi-structured interviews by telephone with eight individuals with GVHD between May and August 2019. All interviews were recorded using an Olympus DSS digital recorder. Each interview was approximately 1 h long and consisted of two sections. Participants were each given a payment of £50 following completion of the study interview to cover any incurred expenses. A priori themes informed by the initial results of our prior systematic review [7] were used to develop the interview schedule (“Appendix 1”). In the first section, the effect of GVHD on QOL was explored. In the second section, patient experience of the healthcare service provision was investigated, including the multidisciplinary clinics and any support services used by participants. Recordings of the interviews were transcribed verbatim by a professional transcription service and anonymised. A sample of each transcription was compared to the original audio recording to ensure accuracy.

### Analysis

A qualitative researcher (IDVH) undertook a qualitative thematic analysis on eight full interview transcripts, using a constant comparison and mind mapping approach [22]. The qualitative analysis software NVIVO 12 PLUS was used to manage and code the interview data. There was no pre-identified theoretical framework; all codes were derived directly from the data. Data immersion was completed (IDVH) by reading the full transcripts and coding three transcripts. A draft coding manual was developed and modifications made to the coding manual. Contradictions and *‘negative cases’* were actively sought. Once coding of data was complete, the analysis team (IDVH and JK) discussed and refined themes from the codes derived from data. Triangulation within the team provided different interpretations and perspectives to expand understanding of data. The Standards for Reporting Qualitative Research (SRQR) guideline [23] was followed in the write-up process.

## Results

Eight patients consented to participation in the study with mean time post-transplant 3 years at time of interview (see Table 1 for participant demographics). There was a range of disease severity and types of symptoms amongst participants, although we did not formally categorise severity, as the focus of our study was subjective patient experience. All participants had experienced chronic GVHD, however, they did not clearly distinguish in their accounts symptoms that might have been part of an acute GVHD picture.

**Table 1:** Participant demographics

Age (years)	
Mean (range)	60 (46–68)
Sex, *n*	
Male	6
Female	2
Ethnicity	
White	7
Asian	1
Occupation	
Project manager	1
Retired	3
General practitioner	
Project manager	
Teaching administrator	
Receptionist	1
Unable to work due to symptoms	1
Unable to work due to immunosuppression	1
University academic	1
Time since transplant, years	
Mean (range)	3 (0.25–15)
Initial diagnosis	
Follicular lymphoma	1
Myelodysplastic/myeloproliferative neoplasm overlap	1
Myelodysplastic syndrome	2
Acute myeloid leukaemia	1
Multiple myeloma	1
Acute lymphoblastic leukaemia	1
Peripheral T-cell lymphoma	1

Data analysis addressing QOL issues in patients with GVHD (*‘Q’*) identified five core themes, and data analysis addressing patient experience of the healthcare provision (*‘E’*) identified a further four themes: see Table 2 for a list of key themes.

**Table 2:** Key themes related to QOL and service provision

Themes related to QOL issues in GVHD	Themes related to patient experiences of the healthcare service provision
*Q1: ‘Restricted as to what I can do’*	*E1: Personal care and close relationship with BMT nurses*
*Q2: Troubling symptoms—‘you can sort of get GVHD anywhere’*	*E2: Efficiency versus long waits—‘On the case straight away’*
*Q3:* *Confusion and uncertainty regarding GVHD symptoms—‘Is this the GVHD?*	*E3:* *Information provision—‘went into it with a bit of a rosy view’*
*Q4:* *Unpredictable course and uncertainty regarding the future*	*E4: The role of support groups*
*Q5: Adapting to the sick role*	

### Themes related to QOL issues in GVHD

#### Theme Q1: ‘Restricted as to what I can do’

A recurring theme was that GVHD is a life-altering condition, with significant implications for an individual’s day-to-day life. There was a notable spectrum of GVHD disease severity, ranging from individuals who reported times where they were fully bed-bound with their GVHD (P6, P8), to those who made simple but significant alterations to their behaviour, for example avoiding sun exposure which could trigger GVHD of the skin (P3). One participant (P7) reported that her GVHD did not restrict her at all, aside from the time taken to apply topical ointments for her cutaneous disease. Another participant (P2) described the effect directly imposed by her ‘*plethora of medical appointments’*, explaining how *‘instead of doing… done all the things that we would have done in retirement, we're now shuttling backwards and forwards to Oxford.’* References to the negative psychological impact of not being able to carry out their usual daily activities were made, with one participant explaining *‘it’s quite frustrating that I can’t do the things that I want to do because I get short of breath and fatigue and nausea’* (P5).

Half of the patients did not distinguish between restricting activities because of their HSCT [for example, *‘immunosuppression means I can't mix in the same way as I did before’* (P2); *‘I haven't been able to be in contact with grandchildren because of infection risk*’ (P3); restrictions on sports such as not being able to go *‘swimming… mainly due to the risk of infection’* (P5); and restrictions regarding public transport: *‘I wouldn’t be able to hack a trip into London to say nothing of going on public transport with a risk of infection’* (P8)] from those imposed by the effect of having GVHD itself. We have focussed on examples that are considered to be illustrative of restrictions imposed directly by having GVHD rather than the transplant itself, but importantly this seems to be a somewhat artificial distinction when imposed upon several of the participants’ stories. Illustrative quotations are presented in Appendix 2, Table 3.

#### Theme Q2: Troubling symptoms—‘you can sort of get GVHD anywhere’

Participants cited examples of a variety of different ways that GVHD had manifested. Cutaneous symptoms (all patients), fatigue (P2, P4, P5 P6, P8), and oral symptoms (P1, P2, P4) were most commonly described, but other symptoms reported included: eye dryness (P1, P2), loss of appetite (P5, P8), tendon pain (P1), ankle and leg swelling (P4) and shortness of breath (P5). Symptoms were also caused by treatment taken for GVHD symptoms, for example insomnia caused by corticosteroids (P5, P6).

There was an overlap specifically between oral symptoms and the restrictions discussed in theme 1, as participants reporting these symptoms described being forced to alter their diet for example avoiding *‘crispy’* or ‘*spicy’* foods (P1). Two participants also described loss of appetite even in the absence of mouth ulceration (P5, P8).

Cutaneous symptoms provoked a variety of different reactions, ranging from embarrassment; frustration with itch or pain; distress from pain; finding treatment application regimes tedious; and the psychological impact of skin symptoms serving as a constant reminder of their GVHD. Although feelings of embarrassment were predominantly associated with skin symptoms (P3, P4), one female participant described feeling self-conscious of singing in her choir because of concern about the appearance in her mouth (P1). One participant (P5) described how his GVHD had resulted in *‘a depression’*, and he alluded to the cause of this being a combination of the actual symptoms, worry about the disease and treatment side-effects—for example stating that he knew being ‘*grumpy*’ was a side-effect of taking oral corticosteroids. See Appendix 2, Table 4 for illustrative quotations.

#### Theme Q3: Confusion and uncertainty regarding GVHD symptoms—‘Is this the GVHD?’

Five of the participants described challenges arising from the non-specific nature of symptoms, with the result that it was often difficult to predict. This unpredictability often provoked anxiety, with every-day aches and pains taking on new significance as potential signs of worsening or spreading GVHD. One participant described how *‘it is just a very strange thing. You just never know, because another little thing, and I'm… 'Mm, could that be it?' you know, cos you just never know where it's going to crop up next really’* (P1).

The diverse nature of possible symptoms also led to diagnostic uncertainty, with two participants reporting consulting with multiple healthcare professionals before confirming a diagnosis of GVHD (P1, P4). One participant described being treated with oral corticosteroids for lung symptoms that *could* be GVHD—to ‘*be on the safe side’* (P5). This sense of ambiguity regarding symptoms also led to frustration over exactly what could be treated: ‘*it was just a case of I was a bit frustrated even more, you know, what the hell's going on, more than worrying about things. It was more a case of just being a bit frustrated that, you know, there's clearly something wrong here, but what it is, can we treat it?*’ (P6). Furthermore, five of the patients had known co-morbidities (participant 3: severe HSV of the tongue; participant 4: chronic lymphoedema presumed secondary to GVHD and dilated cardiomyopathy; participant 5: persistent perianal fistula and haemochromatosis carrier; and participant 7: diabetes mellitus; sensory motor neuropathy and post-menopausal vulval atrophy). This is unsurprising considering the average participant age was 60, but the presence of such co-morbidities might further contribute to diagnostic uncertainty with new GVHD symptoms.

Illustrative quotations are presented in Appendix 2, Table 5.

#### Theme Q4: Unpredictable course and uncertainty regarding the future

The unpredictable nature of the overall disease course was also repeatedly described. This led to uncertainty about the future, with implications for planning ahead with their life plans: *‘it used to be a, you know, to look forward to growing old and everything, but now I just have to… I just literally live one day at a time and just, only look like a few months ahead’* (P4). Frustration was reported because GVHD was a slow or tumultuous recovery process and was chronic: *“I suppose the recovery's been very slow, so there's been a large frustration during the recovery”* (P6).

One participant, who was predominantly affected by painful oral symptoms, described her concern that the GVHD could arise somewhere in the body where it could be ‘*a risk to [her] health’* (P1). However, despite worry and uncertainty about the future, and the anxiety about possible future symptoms described in theme Q3, several participants shared optimism about the future and how their GVHD would progress (P3, P6). This optimism was often cautioned by realism and had the sense of a positive outlook rather than coping through wishful thinking, for example *‘Because I'm quite strong in my mind, I've turned it round and turned the negatives into positives, so I'm back on the… I'm on a slow up, if you know what I mean’* (P4). See Appendix 2, Table 6 for illustrative quotations.

#### Sub-theme Q5: Adapting to the sick role

Several participants described how they found the diagnosis a ‘*shock*’, both in reference to the initial diagnosis which warranted a transplant (P5, P7), but also regarding the development of GVHD symptoms post-transplant (P1, P4, P6). One participant (P2) gave a sense that there was some shift in his self-identity imposed by a new life in which he must constantly take tablets and attend hospital appointments. Another participant (P8) described the difficulties keeping track of constantly changing doses of GVHD medications, which served as a ‘*constant reminder’* of the condition to which he must adapt. The psychological difficulty of adapting to the diagnosis was also implied, with echoes of frustration: ‘*why, what have I done?*’ (P4). See Appendix 2, Table 7 for illustrative quotations.

### Themes related to patient experience of the healthcare service provision

#### Theme E1: Personal care and close relationship with BMT nurses

Personal and attentive care was a key feature that participants described about their experience of the BMT clinics throughout the interviews. The close relationship with their named BMT nurse was the most frequent example provided, with repeated descriptions of the usefulness of having someone readily available to speak to on the telephone (P1, P2, P3, P8), for example, ‘*Oh my gosh, they are wonderful. Because I have a transplant nurse… who I can call … like I called her when something else flared up a little while ago…I was in there and they saw me straight away… If I had lots of questions that I could give someone a call and maybe get some answers or… yeh, or reassurance or whatever.”* (P1). Both the comfort and increased efficiency of speaking to someone who already knows your medical history was also described—as opposed to having to *‘start again’* with a new healthcare professional (P2). One participant (P4) was emphatic about the importance of being treated with attentiveness and *‘a smile’* from all members of the team.

Overall, participants were very positive about the clinical care they had received from the clinic doctors. One individual (P8) described how his consultant physician would personally call him between appointments to check in on dose adjustments or suggest alterations. This participant did have an issue, that was not further elaborated upon, with one doctor that he saw. The participant, however, reported that he spoke to his BMT specialist nurse about the issue, who arranged for him not to consult with the doctor in question again. Another participant (P6) explained that *‘there's always the thing about the logistics…it's always a tricky thing, but in terms of the expertise and the consultants I see, I've got no real issue. It's just that I see lots of different people rather than the same person.’* Illustrative quotations are presented in Appendix 2, Table 8.

#### Theme E2: Efficiency versus long waiting times—‘on the case straight away’

Three participants described examples of how they felt that their problems had been dealt with very promptly (P1, P2, P3). Interestingly, there was some overlap with theme E1, as participants cited examples in which the close relationship that they had with their named BMT nurse facilitated efficient care: *‘I think that it's probably the availability of [named BMT nurse] on the end of the phone that, you know,…you can have a friendly voice that knows you, so I think a personal relationship is important. And the fact that she has access to all of the other colleagues that she can then go and speak to and get back to me’* (P3). In contrast to most accounts of ‘*attentive care’* (P3) and things being dealt with ‘*very quickly’* (P1), one participant (P6) felt that on one occasion he contacted emergency triage services and felt that their failure to act on his concerns led to a delay in his GVHD diagnosis. The convenience of having a dermatologist embedded within the team, enabling streamlining of their GVHD care, was also referenced to by multiple participants (P2, 3, 5).

However, there were also descriptions of clinic delays and long waiting times at the hospital—particularly for blood tests (P1) and pharmacy (P1, P2, P7). Interestingly, these were largely discussed with a tone of sympathy for busy staff, or understanding of other urgent clinical need, rather than frustration: *‘Occasionally, it doesn’t happen very often now, but there are occasions where it is a long wait… But then I know that when I was poorly I had the attention of my consultant… there was no rush if you know what I mean, so I appreciate that there would be someone somewhere in with somebody who's very poorly and is taking a long time more, which I understand’* (P1). One participant (P2) described *‘niggly things’* with technology causing issues in clinic, and technical difficulties with prescribing the correct form of his medication (liquid rather than tablet). See Appendix 2, Table 9 for illustrative quotations.

#### Theme E3: Information provision—‘went into it with a bit of a rosy view’

This was a heterogeneous theme, with some participants feeling that they were given sufficient information (P3, P6, P7) while others would have appreciated more (P1, P2, P4, P5, P8). One participant who had their transplant many years ago felt that the information available now, with ever-increasing online resources, is far more detailed than was previously available (P4). Largely, participants felt that they were not prepared for the GVHD, but with the caveat that *‘I’m not sure anything quite prepares you for it’* (P8). Several participants felt that prior to the transplant, GVHD was ‘*mentioned but not stressed’* (P3), with a greater focus on the actual transplant. Another participant (P6) explained that he thought this initial focus on the actual transplant, with further information about GVHD subsequently, was the right approach. In contrast to the prevailing view, one individual (P5) felt that what he imagined of GVHD was worse than he experienced and therefore would have appreciated more information beforehand as a means of reassurance.

In terms of ongoing provision of information during treatment of GVHD, there was an overlap with the idea of *‘attentive care’* addressed in theme E2. One participant (P5) explained how he is given plenty of reminders about how to manage his condition, for example with repeated reminders of the importance of adequate sun protection. However, another participant (P8) described a concerning time in which he experienced proximal myopathy from corticosteroid treatment, but he had not been informed this was a treatment side-effect, so he found the weakness he experienced very discouraging. He suggested that doctors should offer more information about possible treatment side-effects to prevent unnecessary worry in the event of side-effects occurring. Another participant suggested that having good information about the disease would help patients process the ‘*ups and downs*’ better and be ‘*more proactive about their care’* (P5). See Appendix 2, Table 10 for illustrative quotations.

#### Theme E4: The role of support groups

Many participants discussed how speaking to other people who also had experiences of GVHD had been beneficial (P2, P3, P4, P5), or thought that they would benefit from it (P1). There was a sense of the value of shared experience that cannot be replaced by medical expertise alone. One participant (P3) described attending a support group as a *‘sobering’* and *‘scary’* experience and that his wife in fact *‘found it off-putting and alarming’*, due to the fact that members of the group described symptoms far worse than he had experienced. The same participant did also say, however, that the meeting gave him more confidence regarding the possible treatment options for GVHD. Another patient (P6) did not feel that support groups would be valuable to him, although he was *‘open to it’*, because he already had good support from his family. Illustrative quotations are presented in Appendix 2, Table 11.

## Discussion

Five themes related to the specific QOL issues experienced by individuals with GVHD were identified from our thematic analysis with four themes relating to patient experience of the specialist multidisciplinary BMT service.

A striking feature of the data is the variation in symptoms described and the extent to which these symptoms affect the patients’ day-to-day lives, which reflects the known heterogeneous nature of the condition [24]. Indeed, the concept of the distinct lack of uniformity and unpredictability of the condition was what most united the individual participants’ stories. The ambiguity of cGVHD has also been identified as a key theme in previous qualitative work exploring the psychosocial and emotional impact of cGVHD experienced by patients [11], and in survivors of HSCT more generally [12]. The sub-theme ‘*adapting to sick role*’ emerged from several of the participants’ accounts. We suggest that the unpredictability of the disease course of GVHD makes this adaptation particularly challenging, with constant adjustment of treatment dosing regimens and unpredictability of flare-ups meaning patients have to adapt to a continuously evolving sick role. Indeed, Moss-Morris [25] postulates that *‘self-efficacy/sense of control regarding disease management’* is an important factor in successful adaptation to a long-term condition.

Participants describe highly positive experiences of the care that they receive from a multidisciplinary BMT service, with efficiency, attentive and personal care being cited throughout. The convenience of having a dermatologist embedded within the team, enabling streamlining of patients’ GVHD care, is particularly pertinent when interpreted in parallel to the QOL data, in which some participants cited frequent and time-consuming hospital attendances as a factor restricting their normal lives. The positive healthcare experiences cited in these interviews is in contrast to a U.S focus group in 2005 where *‘betrayal by their medical team (inability to help them)’* was identified as a key theme in cGVHD patients [17], and in focus groups exploring healthcare needs of survivors of HSCT more broadly, participants felt *‘left on their own’* without targeted care for their specialist needs [18]. These reports emphasise the issues patients can face with their healthcare service when living with a *‘difficult-to-treat’* chronic disease and the pertinence of multidisciplinary specialist care. As this is the first qualitative study exploring patient experience of multidisciplinary specialist healthcare provision for GVHD in the U.K., it is difficult to interpret whether this contrast could result from international differences in practices, or simply a difference in the U.K. service we investigated versus those particular services in the U.S.

Dunn proposed as a result of her in-depth phenomenological work exploring the experiences of individuals returning home following allogeneic stem cell transplantation [9] that the development of an Advanced Nurse Practitioner skilled in early recognition of treatment effects would be highly beneficial in the care of patients in their first year post HSCT. In support of this, we identified *‘Personal care and close relationship with BMT nurses’* as a key theme relating to patient experience of healthcare service provision and emphasise the value with which many of the patients placed on the importance of having someone who knew their story readily available to speak to.

Our study’s qualitative methodology enabled the complexity and the unique nature of an individual’s experience with GVHD to be revealed. For example, in descriptions such as frustrations regarding clinic delays or long waits for blood tests or pharmacy, the interview data captures important nuances such as the participants’ understanding of time pressures experienced by their healthcare service. Furthermore, the optimism held by many individuals, even in the face of concerns and anxiety about disease progression, was apparent. Indeed, in a qualitative descriptive study seeking how participants cope with living with moderate and severe chronic GVHD, Driscoll et al. identified *‘Positive Attitude, Gratitude and Mindfulness’* as a key theme [8]. Dunn also found that many patients cited feeling lucky to have received a stem cell transplant and expressed gratitude for being given *‘a second chance’* [9].

In a previous qualitative analysis of interviews with HSCT survivors with moderate to severe chronic GVHD conducted by Fishman et al. [10], patients explained that by seeking information and a greater understanding of their disease, it helped them to gain control over it. This was echoed in our analysis, with participants reflecting on the importance of being well-informed regarding possible treatment side-effects, and that comprehensive information helps patients to process what is frequently a tumultuous disease course. In a qualitative analysis specifically focussed on patient education, Jim et al. reported that late complications post HSCT were often unexpected, and patients felt unprepared for the extent to which GVHD could affect their lives [14], which was also captured in our theme *‘Information provision—went into it with a bit of a rosy view’.* Our theme *‘the role of support groups’* has also been previously described, with patients expressing a desire to connect with other HSCT survivors [10, 12, 14].

### Potential limitations

Participants were all recruited from the specialist GVHD clinic, as this was our group of interest, using a convenience sampling method. There is a potential response bias as patients who felt more positive about their overall experience may be more likely to participate in the study. In addition, the demographics of the patients recruited to the study may not be representative of the entire GVHD population. Our participants were recruited from a single tertiary BMT centre and were generally well-educated and mostly belonged to a high socio-economic class. Ashing-Giwa [26] developed a conceptual model of HRQOL, finding that socioeconomic status is an important determinant of QOL. It is therefore likely that QOL issues in patient groups from less affluent socioeconomic backgrounds will vary and will need further exploration. Recruitment for this study was challenging, as many patients did not want to participate due to the high morbidity of their disease and high burden of living with GVHD.

Another potential limitation of this study is that the interviews were conducted by a doctor, so participants might have felt obligated to give socially desirable responses when asked to critique aspects of their healthcare. This was mitigated for by the fact that the interviewer was an academic trainee not involved in the set-up or running of the clinic, and with whom the patients were not familiar. Participants were also assured at the beginning of the interview that their comments would be anonymised, and that any issues raised during the interview would not be discussed with their care team without their explicit consent.

Only eight participants were interviewed for this study. However, sample size in qualitative research is driven at achieving data saturation—the collection of qualitative data to the point where a sense of closure is attained because new data yield redundant information [27]. Whilst we did not reach complete saturation of new ideas, we increasingly found overlap between issues and nine key themes arose. Indeed, the intention of this study was not to compile an exhaustive list of possible GVHD-related issues, nor describe the experience of every GVHD patient. Instead, our aim was to explore individual experiences of living with and being treated for GVHD, as an initial qualitative exploration of this complex systemic disease. Moser and Korstjens [27] suggest in their frequently cited ‘*practical guidance to qualitative research*’ that phenomenological studies *‘require fewer than 10 interviews’*.

## Conclusions

GVHD is a challenging disease to manage due to the highly heterogenous nature of its presentation, and the varied experience of the disease in patients. Significant QOL issues in this group include feelings of uncertainty about the nature of GVHD and its progression, the struggle to accept new restrictions to daily life and adaptation to the ‘*sick role’* and coping with troubling symptoms. The importance of robust information provision was prominent throughout, with one participant suggesting that it enables a patient to better process the tumultuous course of the disease, and echoing findings in previous qualitative literature that a greater understanding of the disease can help with gaining back a sense of control. This does, of course, have to be carefully balanced with the risk of overburdening individuals with predictions in what is ultimately a highly unpredictable disease, and we emphasise the importance of a nuanced, flexible and personalised approach.

Participants in this study highly valued the multidisciplinary care team, including having a dermatologist embedded within the service, enabling streamlining of their GVHD care. The important role played by BMT specialist nurses who acted as gate-keepers for their care was particularly dominant throughout the interviews. Whilst further qualitative research is needed to develop our understanding of the complex issues, we suggest that BMT specialist nurses have an important role to play in managing the unique QOL issues of GVHD patients. BMT specialist nurses are also likely to promote the provision of high-quality information, and participants stated a preference for speaking to healthcare professionals who were familiar with their story and thus able to offer more personalised advice. With many services increasingly embracing the use of remote consultations and telemedicine, we emphasise the importance of maintaining these invaluable relationships remotely.

## Data Availability

All data generated or analysed during this study are included in this published article and its supplementary information files.

## Appendix 1: Interview schedule

### Introduction

“Thank you for agreeing to this interview, which is being conducted to investigate the quality of life of patients with Graft-versus-Host Disease, also known as GVHD, following stem cell transplant. This interview will last for up to 1 h and will be in two sections. In the first section, we will explore how your quality of life has been affected by a diagnosis of GVHD. In the second section, we will explore how you have found the clinical services that are available for patients with GVHD, including the bone marrow transplant clinics here in Oxford and any support services that you have used.”

1. To begin with, could you please tell me a little bit about yourself, why and when you had the stem cell transplant and when you were diagnosed with GVHD?

### Section A: Health-related quality of life issues of GVHD

“So now that we have discussed a bit of the background, we will move on to the first section of the interview, which focuses on quality of life”.

1. What does the term ‘*quality of life*’ mean to you?
2. Do you think the GVHD has affected your quality of life, and if so, how?
3. Has the way in which GVHD has affected your quality of life changed over time since you were diagnosed?
4. What symptoms have you experienced from the GVHD, and how have these affected you?
5. Have your symptoms changed over time? Have they changed in severity?
6. Other than physical symptoms, has the GVHD had an emotional impact on you?
7. As a result of the GVHD, have you had to make any lifestyle changes?
8. Are there any activities or hobbies that you used to enjoy that you can no longer do or have had to change as a result of the GVHD?
9. Has the GVHD had any impact on your partner (if applicable), family members and other relationships?
10. Has the GVHD affected your work or professional life (if applicable)?
11. Has the GVHD led to any financial problems?
12. How have you found the treatments for your GVHD? Have the treatments affected your quality of life? Have you had any side effects from them?
13. How do you see your future?
14. Is there anything else you would like to tell me before we move on to the next section of the interview?

### Section B: Patient experience of current clinical services and supportive care provision

“Thank you for answering my questions so far, I’d like to now move on to the second section of the interview, which is focused on your experience of the clinical services available for GVHD patients”.

1. Could you please tell me about your experience with the care that you have received for your GVHD?
2. How did you find the bone marrow transplant clinic here in Oxford?
3. Has anything about your care been particularly good?
4. Is there anything that you think could be better about the care you have received to manage your GVHD?
5. Other than the bone marrow transplant clinic, have you had treatment or support for your GVHD from anyone else? This could include your GP, other hospital doctors, physiotherapists, psychologists or complementary medicine practitioners.
6. Are there other support services that you think should be available to GVHD patients?
7. Is there anything else you would like to talk about in terms of care that is currently provided for GVHD patients in this region?

### Close

“Thank you very much for your time today in answering my questions about GVHD. Our hope is that this research will make an important contribution to improving the future care of patients with Graft-versus-Host-Disease. If any of the topics that we have discussed today have been difficult or upsetting, then please let me know and we can discuss these issues, and I can offer you options for further support. Do you have any questions?”.

## Appendix 2: Tables of Illustrative Quotations

See Tables 3, 4, 5, 6, 7, 8, 9, 10 and 11.

**Table 3:** Illustrative data extracts from theme *Q1:* *‘Restricted as to what I can do’*

Psychological impact of restricted activities—frustration	“I think that my inability to pursue all of my normal activities has had an impact on my wife, both in the fact that we shared most of those interests and activities, but also psychologically that I'm less tolerant and a grumpier person to be around because of those restrictions.”—P3
	“It's quite frustrating that I can't do the things that I want to do because I get short of breath and fatigue and nausea and things like that.”—P5
Significant impact on activities of daily life	Interviewer: how do you think the GVHD has affected your quality of life? “So, the GVHD completely wrecked it. When I was suffering particularly badly I was literally unable to move much. I work at home a lot, so I was unable to move between my bedroom, my office, which is just, you know, ten/fifteen steps away. I was unable to drive, to do, like I say, basic things around the house, even… My mother came to visit; I was unable to even take her on a short walk around the village, so it really impacted me in terms of my energy levels and my focus levels.”—P6
Restrictions to normal daily activities	“GVHD has changed a lot of my day to day actions in terms of trying to limit sun exposure… I'm sitting around indoors either reading or watching films or doing sort of indoor things that I would never have done in good weather before.”—P3
	“I'd just have to just slow the walk down from being like, like I say, 5 min I used to walk up from town; now it takes me 35 min each way… I've just got to put… add it to my mind wherever I walk basically, whichever way I go; whatever I do.”—P4
	“I was a pretty athletic person. I used to play tennis, football, I used to play pool for my local pub with my best friends and my friends and my brother in law. And I used to drive… driving was my hobby… Whereas now I just… I can barely walk about sort of ten metres and I'm out of breath. I can't do any more sport cos my ankles swell up at the slightest bit when I start walking.”—P4
Restrictions due to treatment side-effects	“Well the Tacrolimus … my hands are very shaky; almost Parkinsonian. It’s been as it's almost impossible for me to do anything handwritten. It makes… means I make a lot of errors on my iPhone when I'm trying to text or send emails on my iPhone.”—P8
Impact on occupation	“My office is normally only 7 min walk away—that’s still a bit too far for me to go. And I have a three-storey staircase to the top of the building where my office is located. And I haven't… you know, I've been too fatigued to do that, to say nothing of the fact that up until relatively recently, as I said earlier, my cognitive bandwidth is severely limited.”—P8
Restrictions due to tiredness or fatigue	“There is days where it’s just no strength and I have to sit and literally get my recliner right up the ankles and as far as I can get it.”—P4
	“I don’t want to over exert myself because if I do that, the next day I'm like completely shattered and I really can't do anything, if you know what I mean; I just feel too tired to actually do anything at all.”—P5
	“For several weeks I was barely able to do much other than lie in, or on, the bed feeling deeply fatigued.”—P8
Adapting to treatment regime	“I can do normal life, except just have to be careful with the sunlight and putting the ointment in the evening.”—P7

**Table 4:** Illustrative data extracts from theme *Q2: Troubling symptoms—*‘*you can sort of get GVHD anywhere’*

Oral symptoms	“Eating is very… is difficult… anything too crispy or crunchy I tend to not eat, or if I do eat I eat it with my front teeth…nice spicy food but can't eat anything spicy anymore… there's something in toothpaste and chewing gum which just burns my mouth if I dare… I have to use a fennel toothpaste… I think my mouth… Has been the worst one I think; has bothered me the most, yeh. Because it's just ongoing and doesn’t seem to ever end.”—P1
	“It started appearing in my mouth, you know my mouth was getting very sensitive, loss of sense of taste, and it's… I mean it was… my mouth was quite sore and ulcerated…it makes eating things like crisps and crunchy toast and stuff like that is a bit problematic …Losing my sense of taste completely has, you know, is a bit of a loss.”—P2
	“I had like a bad ulcer reaction in my mouth; top half of my mouth cos it ended up looking like… the only way I can describe it, it looked like… like a moon sort of crater effect, all the holes is where all the skin and everything got into the… the like ulcer effect on top of my mouth. I had about a hundred odd ulcers we reckon in there once, but that was, as I say, a few years ago. I ended up having to be sort of on a drip sort of thing, and pain and numbness in my mouth.”—P4
Loss of appetite	“She'd [partner] gone to all this trouble to make this really nice meal, and there I am—don’t want… don’t want to eat it, but it was not that at all, it was just the sort of smell of food, the texture, the taste; looking at it just made me feel quite ill.”—P5
	“A loss of appetite; loss of interest in food. And I used to do all the cooking in the household, so it's not that I haven't had an interest in food before.”—P8
Ocular symptoms	“Then my eyes have been badly affected as well, and that’s ongoing; they're still affected. I have… If I cry I have no tears… sometimes they get very, very dry, and it's like sometimes I wake up in the morning, it feels like my eyeballs have stuck to my eyelids where they're so dry; they just don’t have any… they don’t have any moisture in them at all. And they get very sticky as well and gungy.”—P1
	“My eyes are sore, so when I wake up in the morning my eyes are well, hard to open and painful for, you know, probably 2 or 3 min really until I've washed them.”—P2
Cutaneous symptoms	“It was embarrassing because I would have erythrodermic reactions over great chunks of my body if I got hot… if I was out walking I would feel self-conscious about the look of it and, you know, if we're going into a public place it's… although I knew it wasn’t catching you get that impression, so there's a psychological element to the … of the way the skin looks… it's socially embarrassing and therefore, you know, if I catch myself scratching in public or, as I say, when it was florid it was impossible to disguise, so there is the withdrawal from social situations.”—P3
	“Although it's itchy and irritating, it's a painful itch and it can be quite distressing at times.”—P3
	“Severe… it was just very itchy…it was like underneath my breasts and between my legs and just really awful, awful itching.”—P1
	“It was only, you know, a sort of a bit itchy on my head, so it wasn’t like I was walking around, you know, continually wanting to scratch everywhere. You know, it was just sort of there and, you know, and therefore a visible reminder of… of the fact that I had got GVHD.”—P2
	“It was literally like someone had just turned like a dimmer switch on a light; it just literally just went red and it just… it just sort of like grew out… come out of nowhere really. And it was … then it just started getting rather tight and really sort of like, not burny, but really hot like a sunburn effect.”—P4
	“Well it's very tedious having to apply the ointment and then wait 45 min or so, and then put an emollient on and doing it twice a day. It takes a lot of time and it's and very tedious thing to have to do.”—P8
	“GVHD doesn’t really cause me any trouble except because it's the skin; GVHD on the skin.”—P7
Lung symptoms	“And I was told that you could get it… you can sort of get GVHD anywhere, and when they thought it was in my lungs, cos I'd had a lung function test… I think the general consensus now is that it might be something to do with my diaphragm, which stopped working properly. I don’t know, but that was how it felt. And very uncomfortable lying down, if you know what I mean, just for trying to sleep.”—P5
Treatment side-effects	“I think it's not so much the GVHD itself; it's the drugs that go along with it that have caused the…issues.”—P2
	“My sleep patterns have been severely impacted by taking the steroids, so you know, three or 4 h of sleep is… has been typical.”—P6
	“I know that the side-effects are outweighed by the benefits, but it still feels very harsh if you know what I mean? They're very hard on my body and that, where the insomnia in particular, which was very hard because I'd go to bed and I'd be asleep for 40 min or so, then I would just wake up and the only thing I could do would be just to go and watch the tele, and it would be 2–3 h watching television.”—P5
Depressive symptoms	“I was quite surprised at how quickly you can sort of get into this feeling of, you know, of the depression, you know, and it’s quite scary the depression and… Because you don’t… never having been in that situation it was very hard to sort of come to terms with it.”—P5

**Table 5:** Illustrative data extracts from theme *Q3: Confusion and uncertainty regarding GVHD symptoms—**‘Is this the GVHD?’*

Difficulty identifying symptoms	I have a lot of tummy problems and I think, 'Well is that part of graft versus host?' And you think how… without having an op to have a look, how would you know?”—P1
	“I get intermittent gut pain that I'm not really sure why that’s occurring but suspect that it might be some sort of mild manifestation of GVHD.”—P3
	“I suppose the difficult thing… the difficulty I had was that I knew there was something wrong, you know, quite early on, and I remember just saying that at the end of May session with one of the consultants, I knew there was something not right. But GVHD is so hard; you don’t know what it is yourself, so it’s so hard to really put a finger on it, and if you haven’t got an infection or an obvious sign of something wrong with you, you know, like a rash that they tell you about, it can be very difficult itself to understand.”—P6
	“It's difficult to sort of work out what's GVHD and what’s just, you know, mixture of age and lack of practise.”—P2
	“Then about 6 weeks ago/7 weeks ago, I started getting breathing problems, and I had to see the XXX and CT scans and they felt that the cause was GVHD in my lungs, and so they prescribed some high course dosed steroids which … they weren’t sure whether it was but they felt that to be on the safe side they needed to assume that it was GVHD because they didn’t wanted it to get worse.”—P5
	“I look at something and I think… ‘is that GVHD or what or is that just me?' I've had sort of problems recognising it.”—P5
	“Also, it's difficult to sort out, for example, the actual effects of the GVHD from some of the side-effects of the medications.”—P8
Accessing multiple healthcare professionals	“I don’t know what I thought I had, and I went to see a pharmacist; I went and saw my GP. Everyone drew a blank. And then I… and then I suddenly thought, 'I wonder if it is anything to do with graft versus host.’ And … anyway contacted the hospital and went in for an appointment, and yes, it was; it was graft versus host in my mouth. So, and nobody could explain why it had suddenly come back after… what, it was 12 years.”—P1
	“They thought it was lymphoedema, one part of it. So, I went to the lymphoedema clinic and the lymphoedema nurse said, 'No, that’s not lymphoedema, that’s … that could be something else.’ So, when I went back in to see the normal nurse they said it was the GVHD, and I was literally within 2 min sent through, pushed through, put on and I think it was like the next day or 2 days I was being talked about and obviously that was… I was back on the treatment tablets.”—P4
Lack of certainty about cause of symptoms	“It seems to me hard for the doctors to tell as well to be honest. I’ve asked them about the reason for certain kinds of symptoms, and they say, 'Well, to be honest, you know, we see this but we're not really sure why it happens.'”—P8
Seeking other sources to self-diagnose	“But then within I think about a month I started getting some very trivial skin signs. I went off to hear a lecture, so it was given by the dermatologist … but this was just at a meeting in Nottingham that had been organised by the Anthony Nolan Trust. And she talked about the signs and symptoms of GVHD, and I realised then that that’s what I was experiencing and asked to see her in the clinic at my next visit, and she confirmed that I had GVHD.”—P3

**Table 6:** Illustrative data extracts from theme *Q4: Unpredictable course and uncertainty regarding the future*

Slow recovery	“The whole recovery process is … it's up and down from week to week.”—P8
Unexpected disease	“In terms of the bone marrow transplant, then it was a bit of a shock to me to get the graft versus host; I didn’t really know what was going on, and I wasn’t really expecting it.”—P6
Challenges planning the future	“Because the chronic just seems never ending… That’s the only thing that would probably get me down is the fact, you know, how long is it going to go on for.”—P1
	“I am a planner. I like to, you know, sort of sort things out and… but with this it's… you know, make sort of tentative plans…It’s all dependant on, you know, what happens with the GVHD and, you know, nobody can answer that question; we just have to keep ploughing on and seeing what happens.”—P2
Affecting any body site	“It's a strange disease because it can affect so many different parts of the body… You know, when was it ever going to end and where is it going to come up somewhere else next or, and where is it? And sometimes it does… it is a bit of a concern sometimes because I think I don’t want it coming up somewhere where it could really be a risk to my health, you know.”—P1
	“But that was the only frustrating thing in terms of the GVHD diagnosis. It was very… it's so non-specific. I mean I could have it anywhere; it could be in my gut or whatever, my lungs, whatever, skin.”—P6
Optimism about the future	“Hope that my system gradually adapts to the new… my new blood cells and sort of they all get to live together happily instead of fighting.”—P2
	*“*It may be over optimistic; I don’t know, but I'm hoping to keep it under better control.”—P3
	“[I] just see the future as well, this is a blip, we’ve got over it; we know how to get over it in the future if it happens again; hopefully recognise the symptoms.”—P6

**Table 7:** Illustrative data extracts from sub-theme *Q5: Adapting to the sick role*

Consulting with healthcare	“Until I went to the doctors and found I had this MDS/MPN crossover, you know, I hardly ever went to the doctors. So, you know, I was never one for taking pills at any sort of regular basis.”—P2
Diagnosis was a shock	“Like how serious the diagnosis was, like when we were told what the life expectancy was without treatment and the risk of dying and things like that, you know, quite a shock.”—P5
Challenges adapting	“It's very tedious. I get very fed up with having to do the application of the ointment. You know, they keep changing the dosages of Tacrolimus and the prednisolone, and so keeping track of that, you know, I have to remember now… this week how many, you know, how many milligrams of each am I supposed to be taking when … So, and, you know, it's a constant reminder of my condition.”—P8
	“Psychological’s been quite… there's day where you just want to be a person. I just think, you know, 'why, what have I done?'”—P4

**Table 8:** Illustrative data extracts from theme *E1: Personal care and close relationship with BMT nurses*

Access to BMT nurses	“I've had ready access to XXX, the post-transplant nurse co-ordinator, and she’s been very good; seems very knowledgeable and very encouraging… I think that it’s probably the availability of XXX on the end of the phone that, you know, within 24/48 h, but usually, you know, within that working day you can have a friendly voice that knows you, so I think a personal relationship is important. And the fact that she has access to all of the other colleagues that she can then go and speak to and get back to me. So, she has been really helpful as an access to information and just practical, you know, prescriptions and things like that.”—P3
	“They spent … spent some considerable time with me when I go in for my clinic appointment, they're always… you know, they're available during working hours on the phone, they're perfectly happy to call up; they will follow-up after the clinic appointments. They are sympathetic, well informed.”—P8
	“Having those individual contacts rather than, you know, just ring up and you'll get a team cos it happens every time. You know, cos otherwise… you know, if you have that, you know, every time, you know, you're explaining your problem and your history over and over again, whereas…the individuals get to know you and remember your history.”—P2
	*[BMT nurses]* “They're very good friends now; they're all friends with my mum and my dad and all my family now as well, cos obviously they’ve been there, seen me at my lowest…If I could give them more than ten out of ten I'd give them a full on eleven… They always put on a smiley face even when you know they're going to be absolutely—excuse my French—bloody knackered… I don’t know to word it, but it be that, you know, that side of them; everyone of them—doctors, nurses, every one of them; even the people that come in, you know, to change your bed, and even the trainees, they're always smiling.”—P4
	“You know, as far as sort of care and attention is concerned from the people, you know, I can’t fault it really.”—P2
Close monitoring	"I've had situations where one of the consultants has called me several times after the clinic appointment once he’s had a look at my bloods and things to suggest that we alter the medications before the next clinic…in fact one of them phoned me only this morning to make sure that an adjustment be made to the meds yesterday was going OK.”—P8
Freedom to raise concerns to BMT nurses	“All but one of the doctors that I've seen I’ve been really very comfortable with and impressed with. The one that I wasn’t particularly enamoured of I mentioned to the bone marrow transplant team nurse, and I have not been scheduled to meet with that particular individual again.”—P8

**Table 9:** Illustrative data extracts from theme *E2: Efficiency versus long waiting times—**‘On the case straight away’*

Rapid care	**“**Every time I have been, and they’ve discovered I’ve got graft versus host somewhere else, or whatever, or even from the first time when I went with my tendon, they’ve been on it very quickly…I can call them, I can send in photos of bits of me that I think are affected, and they're on the case straight away.”—P1
	“I've had regular outpatients follow-up and very attentive care from the consultants and their colleagues. They're always very keen and helpful in terms of prescriptions that they're… have prompted, you know, my need for things and pre-empted my need for prescriptions.”—P3
Efficient clinics	“The bone marrow transplant clinics in the… at the Churchill… I tend to go and get seen quite quickly.”—P1
	“I've been impressed basically in terms of, you know, efficiency… it all seems to be, you know, well organised, efficient, or reasonably efficient when I go, you know, you're never seen exactly on time but, you know, you do get seen… people do spend time with you, you know, the nurses are very helpful, you know, always willing to, you know, answer your questions, respond, help where they can. Yeh, and it seems to… well, from my point of view it seems to work quite well; very well, not just quite well; very well.”—P2
Dermatologist available	“I think that the [hospital] has been good in terms of having, you know, a dermatologist on the team.”—P2
	“The once that I asked to see a dermatologist I was able to see her that same day, so that was reassuring.”—P3
	“I've seen a dermatologist here who was able to treat me there because I got it in my scalp; on my scalp as well, so she was able to prescribe lotions for that, rather than, you know, like the Eumovate and things like that, and also recommend shampoos that were a good source, sort of keep them down and things like that. And she was excellent.”—P5
Lack of efficiency	“I think the only thing I would have said is that, that maybe … maybe at that stage where I did contact the triage, maybe could have… we were proactive; we did a blood test, but I didn’t feel that the follow-up was that proactive, it was just a case of well, come to clinic, they didn’t… they had the results already; they knew there was something wrong with the liver enzyme rising, but it wasn’t something they wanted to action straight away.”—P6
	“And we're usually out of those, pending on how long the queue is for blood tests, and then if I have to get any medications from the little pharmacy ‘cos that can be a long wait as well sometimes. I would say on average about two and a half/three hours altogether.”—P1
	**“**You know, the pharmacy always takes… seems to take forever.”—P2
	“It's all around the things like IT, you know, like there doesn’t seem to be any system for recording, you know, what drugs you're on, and you know, therefore, you know, when I want a repeat of drugs, you know, I have to… you know, we have to have a discussion and it’s hard to find, you know, certain ones on the system because like I’m having liquid Cyclosporine cos I can use… so, I have it as a mouthwash as well, so that’s unusual, you know, and that’s… you know, always causes a bit of a… problem both for the prescriber and the pharmacy. So, it’s sort of, you know, little niggly things like that that are the issues.”—P2
	**“**Got to wait quite a long time, and then even after seeing the doctor got to wait for pharmacy and things like that. So, it’s the clinic appointment time, it's late, like at twelve or something. It's really waste XXX all day because it's the middle of the part of the day is in the hospital, so it’s really you can't do anything on that day. So, that was a bit difficult, but if the appointment is early morning then well, sort of just after lunchtime it might finish. In that case afternoon we can do whatever we want.”—P7

**Table 10:** Illustrative data extracts from theme *E3: Information provision—**‘went into it with a bit of a rosy view’*

Information does not adequately prepare	“I think I was a bit naïve because I was… I had focused more on what could happen straight after the transplant…And I think I was just so pleased I'd completely forgotten about what could happen later, yeh. So, it was… it was a little bit of a shock… I think I was more told about what could happen straight afterwards. So, I was told the worst scenario is when I'd had… after I'd had the transplant, rather than what could happen further down the line, yeh. There was more focus on that.”—P1
	“Well, in general terms I was told this was going to happen, but I'm not sure anything quite prepares you for it.”—P8
	“I don’t know whether I got the wrong impression or perhaps didn’t understand it quite enough, but I didn’t… I didn’t think that the after effects of the transplant would last quite so long. I think that’s… I mean it wouldn’t have made any difference to my decision to go ahead. I mean that decision for me to go ahead and have the transplant cos essentially there was, you know, no other option… I probably went into it with a bit of a rosy view of how long the after effects would last, and perhaps not fully understood what those after effects could be. But the trouble with all that is, you know, you're on a spectrum and the poor old medics don’t know where you are until they’ve… on that spectrum until they’ve done it. And you're not going to find out until you’ve had it done, you know, they can spout statistics at you, but in the end, you know, you don’t get a… you don’t get fifty percent rash.”—P2
Sufficient information at the right time	“I think that it was certainly mentioned but not stressed, and I think that I probably had sufficient information. I think once I had had the transplant then interestingly, I was given the book; I think it's called Seven Steps or something, which is the Anthony Nolan guide, which goes into it into a lot detail. So, in some ways I feel a bit as though I was given the small print after the event and the broad brushstrokes beforehand.”—P3
Lack of information	“I think, me personally, I could have done with a little bit more information, but it might have been cos I didn’t know what to ask or sort of what, you know, fully to expect… Whereas, now it's, you know, you can… there’s loads of stuff online you can read and find out and they give you the information as they’re all bit more… bit more on the ball so to speak.”—P4
	“I think maybe we could have had a little bit more of an explanation as to what it is and what causes it, and what was like the treatments that are about that we could have… that we would be given… I think we could have been given more information just as to what to expect, cos I was expecting, you know, like the … I thought I was going to end up looking like, you know, blobs and lumps and bumps… In actual fact, it wasn’t as bad as I thought it was going to be, but I really thought that I was going to look hideous…They told me everything that was going to happen to me when the stem cells went in, and they were right about that, but that little bit afterwards I think I could have maybe done with a little bit more information just to … to sort of like put my mind at rest as to know what to expect because it was an unknown quantity.”—P5
	“We were told that there would always… we’d always be ups and downs and it's great when you’re on an up, but when you’re on a down you know, it’s sort of a… and you don’t understand why, is quite depressing. But with more information to understand why it’s happening I think that the people would be… they would be more proactive about their care, and it would be easier for them.”—P5
Sufficient information given	“Fortunately, they gave us a very good book—the Bloodwise book, which explained it in a way that it didn’t scare the life out of you if you end up going on Google or something like that.”—P5
	“So, the information I was given, the leaflet or booklet like GVHD, was very good and I read carefully, and if I don’t understand or have a question, so I ask the doctor and things like that. So, didn’t really much problem I had.”—P7
	“I did have enough information but I think it's … it’s human nature to listen and understand the information rather than really taking it in and understand the implications of it, and I was certainly guilty of that. I mean I read all the books. They told me what it can do, and it can last for a long time… so yeh, I was well prepared, there was good sessions from the nurses beforehand, but I think on balance it was the right thing… way of doing it because I think you should focus a lot on the process of going through the bone marrow transplant itself and the early days of recovery rather than necessarily focus on GVHD.”—P6
Consistent reminders in clinics	“[Staff in the clinic make] every effort to make sure that I'm aware of like the problems… so, we are aware of where we're coming from in that respect… The nurses that I see in the clinic and the consultant, they’re always looking… always checking you out… checking you out that they sort of see how well it’s doing and that, and they advise us accordingly to what we should be doing, which is good.”—P5
Side-effect not clearly explained	“When I went on the oral steroids…nobody explained to me in advance that one of the side-effects of that was weakening of the musculature in the pelvis and the thighs, and I got quite discouraged by the fact that I seemed to be getting worse at climbing stairs and walking than I had been before. But once I realised it was a drug side-effect that was clearly a relief.”—P8

**Table 11:** Illustrative data extracts from theme *E4: The role of support groups*

Seeking a support group	“It would be really good if I could meet other people that were mainly in the same boat that I’m in, like a little support group maybe for graft versus host disease, you know, so we can all sort of like see if anybody else has got the same similar things that I've got, and we can compare notes.” (P1)
	“I think that it may be helpful for other non-medical patients to have access to more patient self-help support. So, I think that Anthony Nolan do provide a lot of link ups with other patients, and I think that could be stressed more and I don’t know whether it was because I, you know, that wasn’t stressed to me and I've just stumbled across it, but it's there in the literature but I think because staff provide such a good service that … themselves, that they don’t just push you on to somebody else. But actually, it might be helpful for some people to … to be able to talk with other, you know, expert patient people who’ve been that way before and things like that.” (P3)
Value of a support group	“I have actually because in [local town] there's a blood cancer work group which I have been to one of their meetings. And there were other people there who had had sort of similar reactions, and it was nice to talk to them; to somebody who was… has had the same problems as you. Because if you’ve never had it it's very hard to talk to a non… or say to a person who've not got medical training because they sort of like don’t really know, do you know what I mean?” (P5) After going to a support group: “I should ask to go and see a dermatologist because, you know, I've got skin problems which are not being dealt with.”—P2
Negative aspects of a support group	Interviewer [re experience of a support group]: Did you find kind of hearing from other patients useful? “Well that was quite scary in a way because I went on that thing just about 6 months after my transplant, and everybody said, ‘Ah yes, the first six months are easy and this is where it will all go wrong,’ and true enough it did… I think my wife… found it off-putting and alarming… But and it also gave me the confidence that there were other treatment modalities available. But it's… it was a sobering experience to hear not just the technical details of what could go wrong, but to see people that were experiencing those problems.”(P3)
Online sources of support	“Probably most of its probably online to the Anthony Nolan and Maggie's; there's stuff on there. But because I've done a few sort of… sort of helped a few other people with speaking about my experience. So, I can always email people and they always send me all the latest stuff; the up-to-date stuff, so it is online now. But I get… I generally, when I pop up the hospital I always pop into Maggie's and ask them if they’ve got anything, and they always give me links to find stuff. So, it's all online; it’s just having to look for it.” (P4)
Strong family/friends support	“I'd be open to it, but I'm also not really bothered by that cos the one thing I have realised in the whole of this past few years is that, to be honest everyone is different and it’s best focusing on what you're going through and how you deal with it, and if you want to talk to people there's plenty of support groups out there. I just haven't reached out to them; I get good support from my family.” (P6**)**

## Abbreviations

GVHD: Graft-versus-host disease
aGVHD: Acute graft-versus-host disease
cGVHD: Chronic graft-versus-host disease
HSCT: Haematopoietic stem cell transplantation
PROMs: Patient-reported outcome measures
QOL: Quality of life
